# Strategy for acute DeBakey type I aortic dissection considering midterm results: a retrospective cohort study comparing ascending aortic replacement and total arch replacement with frozen elephant trunk technique

**DOI:** 10.1186/s13019-024-02484-6

**Published:** 2024-01-22

**Authors:** Sho Takagi, Yoshihiro Goto, Junji Yanagisawa, Yui Ogihara, Yasuhide Okawa

**Affiliations:** Department of Cardiovascular Surgery, Toyohashi Heart Center, 21-1 Gobudori, Oyama-Cho, Toyohashi, 441-8530 Japan

**Keywords:** Frozen elephant trunk technique, Aortic dissection, Ascending aortic replacement, Total arch replacement

## Abstract

**Background:**

Acute type A aortic dissection is treated with an emergency procedure that uses ascending aortic replacement (AAR). However, to avoid a residual dissected aorta with a false lumen, total arch replacement (TAR) is required. The frozen elephant trunk (FET) technique is a promising surgical approach that promotes false lumen obliteration in a single step. Therefore, this retrospective single-center study aimed to evaluate the operative outcomes of AAR and TAR with FET.

**Methods:**

Between 2007 and 2021, 143 patients with acute DeBakey type I aortic dissection underwent a central repair using AAR (n = 95) or TAR with FET (n = 43). All perioperative variables, the duration of all-cause mortality, and aortic events defined as dilatation of the distal aorta > 5 cm, new occurrences of aortic dissection, distal aortic surgery, and distal aortic rupture were recorded. We compared these perioperative variables and mid-term results with an additional focus on distal aortic events.

**Results:**

Patient background data did not differ between the two groups. Perioperative results for the TAR with FET group vs the AAR group showed similar operative times (306 vs 298 min, P = 0.862), but the TAR group had longer cardiopulmonary bypass times (154 vs 179 min, P < 0.001). The freedom from all-cause death for the TAR vs AAR groups using the Kaplan–Meier method was 81.9% vs 85.4% and 78.0% vs 85.4% (P = 0.407) at 1 and 3 years, respectively. Freedom from aorta-related events was 90.6% vs 97.6% and 69.3% vs 87.0% (P = 0.034) at 1 and 3 years, respectively.

**Conclusions:**

TAR with FET had comparable perioperative results to AAR in acute DeBakey type I aortic dissection and was considered a valuable method to avoid aorta-related events in the midterm.

## Background

Acute type A aortic dissection (ATAAD) is a complex and life-threatening cardiovascular disease; aortic rupture and cardiac tamponade are the leading causes of mortality. Without emergency surgery, the mortality rate is approximately 50% within 48 h, so surgical management is immediately required to prevent mortality [1, 2]. In the surgical management of ATAAD, ascending aortic replacement (AAR) or hemiarch replacement are often deemed sufficient, because a total arch replacement (TAR) is considered more invasive. Approximately 85% of patients with ATAAD have had a limited aortic resection, such as a hemiarch replacement [3]; therefore, emergency surgery using a simple AAR may be acceptable to prevent death. In contrast, TAR is required to avoid a residual dissected aorta with a patent false lumen. However, this procedure carries increased risks due to the extensive aortic replacement and longer surgical duration, increasing the chances for early and late in-hospital death [4]. The treatment of ATAAD is controversial, particularly when the aortic arch and descending aorta are involved [5]. However, the frozen elephant trunk (FET) technique shows promise as it enables simultaneous obliteration of the false lumen without significantly increasing operative risk [6]. In patients with ATAAD, TAR with FET yields more favorable postoperative aortic parameters compared to procedures without FET [7, 8]. Few studies have compared AAR and TAR perioperative outcomes, particularly regarding distal aortic events [9]. Moreover, no reports have compared the perioperative results and prognoses of AAR and TAR with FET. In this study, we compared the perioperative and mid-term results with an additional focus on distal aortic events in patients with ATAAD who underwent AAR or TAR with FET.

## Methods

### Data source

**Fig. 1:**
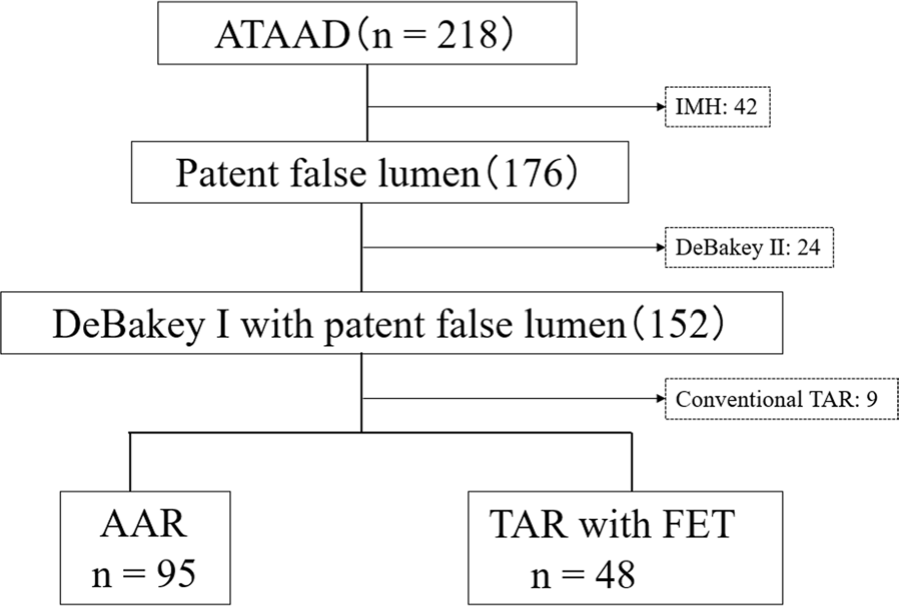
Patient flow diagram. Between 2007 and 2021, we performed central repairs within 24 h for ATAAD in 218 patients included in this study. We excluded patients with intramural hematoma (n = 42), DeBakey type II (n = 24), and conventional TAR (n = 9). We retrospectively reviewed patient data from 143 patients’ medical records. ATAAD, acute type A aortic dissection; IMH, intramural hematoma; AAR, ascendingaortic replacement; TAR, total arch replacement; FET, frozen elephant trunk

This study aimed to compare the perioperative and mid-term outcomes of patients with ATAAD who underwent AAR or TAR with FET, focusing on distal aortic events. The study population consisted of 218 patients who underwent central repairs for ATAAD within 24 h from onset at the Toyohashi Heart Center between 2007 and 2021. In 2017, our hospital implemented TAR with FET, instead of AAR, as the standard procedure for DeBakey type I aortic dissection, regardless of the entry approach. Patients with intramural hematomas (n = 42), DeBakey type II lesions (n = 24), and who underwent conventional TAR (n = 9) were excluded from the study. Before 2017, conventional TAR had been performed at the surgeon’s discretion to avoid the increased risk in very young patients and rapid loss of consciousness due to dissection in the cervical branch. The medical records of 143 patients were retrospectively reviewed (Fig. 1). Patient anonymity was maintained, and the study was conducted in accordance with the principles of the Declaration of Helsinki. The Ethics Committee of Toyohashi Heart Hospital, Aichi Prefecture, approved the study protocol, and written informed consent was obtained from each patient (approval number 220309).

### Study variables

Patients were dichotomized into two groups: those who underwent AAR, including hemiarch replacement (n = 95), and those who underwent TAR with FET (n = 48). We examined both groups’ preoperative conditions, characteristics, operative findings, and midterm outcomes. The duration of all-cause mortality and aortic events was assessed. Aortic events were defined as dilatation of the distal aorta > 5 cm, new occurrences of aortic dissection, distal aortic surgery, and distal aortic rupture.

### Operative techniques

Since the study lasted an extended period, the operative techniques changed throughout. The operative techniques used are detailed below.

Median sternotomy was performed on all patients as the surgical approach. The choice of arterial cannulation site predominantly involved the femoral artery on one side, with occasional utilization of the axillary artery on one side.

### Ascending aortic replacement

The sites of venous cannulation were the superior and inferior vena cava for retrograde cerebral perfusion. Cardiopulmonary bypass (CPB) was initiated, and the patients’ temperatures were lowered to 25 °C. Under circulatory arrest, the aorta was opened, and retrograde cerebral perfusion was established. The aorta was then transversely dissected, proximal to the origin of the brachiocephalic artery. A paired Teflon-felt strip was applied inside and outside the aorta to reinforce the aortic stump. After performing distal anastomoses to the one-branched J graft (Japan Lifeline, Tokyo, Japan), rewarming commenced via systemic perfusion from a grafted branch. Proximal anastomosis was completed during warming.

### Total arch replacement using the frozen elephant trunk technique

Venous cannulation was performed at the right atrium for CPB. Patients’ temperatures were reduced to 28 °C. During cooling, three brachiocephalic arteries were dissected. Under circulatory arrest, the aorta was opened, and antegrade cerebral perfusion was established through the brachiocephalic and left carotid arteries. Transverse dissection of the aortic arch was performed between the left subclavian artery (LSA) origin and the left carotid artery (LCA). The FROZENIX FET graft (Japan Lifeline, Tokyo, Japan) was inserted and deployed in the true lumen. The aortic stump was then externally covered with a Teflon felt strip, and the FET graft was positioned internally. Following distal anastomoses using the four-branched J graft (Japan Lifeline, Tokyo, Japan), rewarming commenced via systemic perfusion from a grafted branch. During warming, the proximal anastomosis was completed. After the proximal anastomosis, aortic declamping allowed for coronary artery reperfusion. The subsequent step involved connecting the LSA, LCA, and brachiocephalic arteries to their respective graft branches through anastomoses.

### Statistical analyses

Univariate comparisons of patient characteristics and operative results according to the range of replacement were summarized using numbers and percentages for categorical variables and medians and interquartile range (IQR) for continuous variables. Categorical variables were compared using Fisher’s exact two-tailed test.

The follow-up duration was until the patient’s death or the last known survival date. Event-free and survival rates were calculated using Kaplan–Meier estimates, and the differences between the two groups were assessed using log-rank tests.

A P-value < 0.05 was considered statistically significant. All statistical analyses were performed using IBM SPSS Statistics for Windows, version 22. (IBM Corp., Armonk, NY, USA).

## Results

The 143 patients were grouped according to the type of surgery, AAR, or TAR with FET (Table 1). We found no significant baseline differences in age, sex, preoperative cardiac tamponade, malperfusion, consciousness disturbances, and hemodialysis history between the groups. Malperfusion resulted from end-organ ischemia including the coronary, carotid, visceral, renal, and lower extremity branch vessels in the setting of an aortic dissection, and was diagnosed based on patients` symptoms, electro-cardiogram, and computed tomography angiography.

**Table 1:** Differences in patient characteristics according to the range of replacement

	All patients (n = 143)	AAR (n = 95)	TAR with FET (n = 48)	P-value
Age, years	63 (53–71)	65 (54–71)	60 (52–70)	0.16
Male (%)	91 (63.6)	58 (61.1)	33 (68.8)	0.366
Cardiac tamponade (%)	12 (8.4)	9 (9.5)	3 (6.3)	0.511
Malperfusion (%)	26 (18.2)	18 (18.9)	8 (16.7)	0.738
Disturbance of consciousness (%)	3 (2.1)	2 (2.1)	1 (2.1)	0.993
HD (%)	3 (2.1)	2 (2.1)	1 (2.1)	0.993

AAR, ascending aortic replacement; TAR, total arch replacement; FET, frozen elephant trunk; HD, hemodialysis

**Fig. 2:**
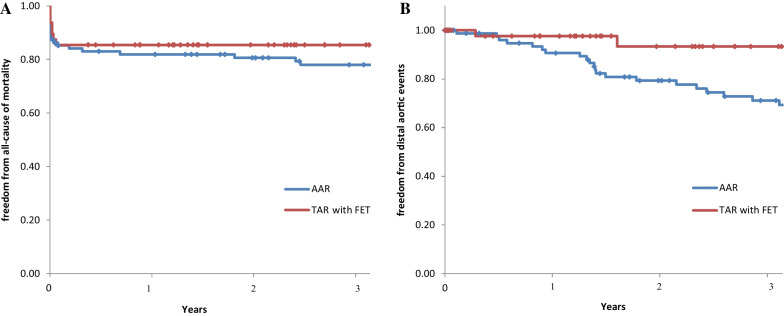
Kaplan–Meier curves after the central repair operation for ATAAD. **A** Freedom from all-cause mortality; **B** freedom from distal aortic events. AAR, ascending aortic replacement; TAR, total arch replacement; FET, fozen elephant trunk

Table 2 depicts operative data and early results. Concomitant root surgery, aortic valve replacement (AVR), and coronary artery bypass grafting (CABG) rates were not significantly different. Mortality rates were 15.8% and 12.5% in the AAR and TAR with FET groups, respectively. Postoperative stroke, paraplegia, mediastinitis, gastrointestinal failure, renal failure, and reoperation for bleeding rates were not significantly different. The median operative and CPB times were 306 min (IQR, 240–395) and 154 min (IQR, 125–186) in the AAR group and 298 min (IQR, 270–349) and 179 min (IQR, 159–200) in the TAR with FET group. The median operative time was not different between the groups (P = 0.691). The median CPB time was significantly longer in the TAR with the FET than in the AAR group (P < 0.001). Hospital stays were 19 days (IQR, 13–28) in the AAR group and 16.5 days (IQR, 10–23) in the TAR with FET group. Freedom from all-cause death and freedom from aorta-related events in the two groups are shown in Fig. 2A, B. Freedom from all-cause death was 81.9% at one year and 78.0% at three years in the AAR group and 85.4% at one year and 85.4% at three years in the TAR with FET group. Freedom from aorta-related events was 90.6% at one year and 69.3% at three years in the AAR group and 97.6% at one year and 87.0% at three years in the TAR with FET group; the difference between the two groups was significant (P = 0.034). Late distal aortic events, defined as events at distal sites over the range of replacement including the abdominal aorta, are shown in Table 3. Distal aortic events were counted based on the first event encountered by the patients. Dilation of the residual aorta was diagnosed in 28 patients after AAR and two patients after TAR with FET. New occurrences of aortic dissection and distal aortic rupture occurred in only one case each in the AAR group. Distal aortic surgery, including thoracic endovascular aortic repair, was performed in eight patients with AAR and three with TAR with FET. Thus, the rate of re-intervention was 8.4% after AAR and 6.2% after TAR with FET.

**Table 2:** Operative data and early results according to the range of replacement

Concomitant	All patients (n = 143)	AAR (n = 95)	TAR with FET (n = 48)	P-value	
Root surgery (%)	11 (7.7)	10 (10.5)	1 (2.1)	0.074	
CABG (%)	11 (7.7)	10 (10.5)	1 (2.1)	0.074	
AVR (%)	5 (3.5)	3 (3.2)	2 (4.2)	0.756	
Operation time (min)	299 (245–383)	306 (240–395)	298 (270–349)	0.862	
CPB time (min)	163 (136–195)	154 (125–186)	179 (159–200)	< 0.001	
Hospital mortality (%)	21 (14.7)	15 (15.8)	6 (12.5)	0.599	
Stroke (%)	22 (15.4)	16 (16.8)	6 (12.5)	0.497	
Paraplegia (%)	5 (3.5)	2 (2.1)	3 (6.3)	0.203	
Mediastinitis (%)	3 (2.1)	3 (3.2)	0	0.213	
Gastrointestinal failure (%)	2 (1.4)	1 (1.1)	1 (2.1)	0.62	
Renal failure (%)	3 (2.1)	1 (1.1)	2 (4.2)	0.22	
Reoperation for bleeding (%)	5 (3.5)	4 (4.2)	1 (2.1)	0.513	
Hospital stay (days)	18 (12–25)	19 (13–28)	16.5 (10–23)	0.157	

AAR, ascending aortic replacement; TAR, total arch replacement; FET, frozen elephant trunk; CABG, coronary artery bypass grafting; AVR, aortic valve replacement; CPB, cardiopulmonary bypass

**Table 3:** Breakdown of late aortic events

	All patients (n = 143)	AAR (n = 95)	TAR with FET (n = 48)
Follow-up time (years)	4.0 (2.3–4.7)	3.2 (1.7–5.2)	2.9 (1.7–4.2)
Dilation of the residual aorta	30	28	2
New occurrence of Aortic dissection	1	1	0
Distal aortic rupture	1	1	0
Distal aortic surgery (%)	11 (7.7)	8 (8.4)	3 (6.2)
Total	43	38	5

AAR, ascending aortic replacement; TAR, total arch replacement; FET, frozen elephant trunk

The diameter of the FET was determined by preoperative computed tomography angiography (90% of the descending aortic diameter at the level of the main pulmonary arteries). Distributions of the size, stent length, and distal end level of the FET used are shown Table 4. We typically used a short FET to prevent paraplegia except in cases where the FET could close the entry of the descending aorta. Postoperative paraplegia occurred in three patients (6.3%), distal stent graft-induced new entry (d-SINE) occurred in two patients (4.2%) requiring thoracic endovascular aneurysm repair (TEVAR). One patient required surgical intervention for a proximal anastomotic pseudoaneurysm.

**Table 4:** FET details (n = 48)

Variables	Result
Diameter, n (%)	
21 mm	1 (2.1)
25 mm	13 (27.1)
27 mm	13 (27.1)
29 mm	12 (20.1)
31 mm	6 (12.5)
33 mm	3 (6.3)
Stent length, n (%)	
60 mm	32 (66.7)
90 mm	12 (25)
120 mm	4 (8.3)
Level of the distal end, n (%)	
Th4	1 (2.1)
Th5	20 (41.7)
Th6	15 (31.3)
Th7	10 (20.8)
Th8	1 (2.1)
Th10	1 (2.1)

FET, frozen elephant trunk

## Discussion

ATAAD is a life-threatening condition requiring emergency surgery to prevent death due to rupture, tamponade, severe aortic valve regurgitation, or malperfusion. In the past two decades, the FET technique has become a valuable and attractive option for treating aortic diseases when the arch and thoracic aorta are involved, both in elective and emergency settings. The FET technique offers the possibility of definitive treatment for combined and extensive lesions in a single-stage procedure [10]. The indications for the FET procedure include conditions such as type A and B, acute and chronic, aortic dissections, and atherosclerotic aneurysms involving the aortic arch and descending thoracic aorta [11].

This study investigated perioperative data, postoperative survival rates, and freedom from aorta-related events in AAR and TAR with FET groups in patients with DeBakey type I. The CPB time was longer in the TAR with FET group compared to the AAR group, but the median operative time showed no significant difference. While TAR typically requires a longer operative time than AAD, using the FET technique allows distal anastomosis to be performed within a similar time frame [7, 12]. Moreover, due to its procedural complexity, TAR is generally associated with a longer surgical duration. However, the FET technique enables the completion of distal anastomoses in a single step [7, 10], making it comparable to AAR in terms of operative time.

Moreover, during TAR with FET, the surgeon can stop intra-operative bleeding more easily than during AAR alone. FET allows for a single-step distal anastomosis, potentially reducing operative time and facilitating bleeding control. In addition, the branched graft used consecutively after FET makes it easier to stop bleeding than an unbranched graft. Furthermore, FET promotes false lumen obliteration, which may improve patient outcomes by reducing the risk of aorta-related events [13].

The extent of aortic replacement during the initial surgery for ATAAD depends on various factors, including the location and extent of the dissection, involvement of vital structures, and the patient’s overall condition. Joon et al. showed that TAR is associated with more significant morbidity and mortality than hemiarch repair in patients with acute DeBakey type I aortic dissection [14]. Other studies have reported negative findings regarding TAR [15, 16]. However, we found no significant difference in mortality between the two groups, with mortality rates of 15.8% and 12.5% in the AAR and TAR with FET groups, respectively.

Survival analysis revealed that the TAR with FET group exhibited significantly higher rates of freedom from aorta-related events. In a separate study, there were no significant differences in the rates of aortic reoperation or dilatation between TAR and hemiarch repair [14]. In contrast, Uchida et al. reported that TAR was linked to a lower occurrence of distal aortic events [9]. Yoshitake et al. demonstrated that the FET technique improved long-term survival rates and the rate of freedom from aortic-related death [13]. Our findings suggest that TAR with FET yields more favorable perioperative outcomes and postoperative aortic events in midterm results than AAR.

A fatal complication of the FET technique is paraplegia caused by spinal cord injury [17, 18]. The mechanism of paraplegia in FET is believed to be multifactorial, and several factors have been identified as potential contributors: (1) intraoperative and postoperative blood pressure; (2) distal position of the stent graft; (3) atheromatous emboli of the spinal cord artery; (4) duration of circulatory arrest; (5) and pathology of the aorta [8, 19, 20]. Recent reports have revealed a lower rate of paraplegia in acute aortic dissection after the FET technique than in atherosclerotic aortas [11]. A stent length of 10 cm is associated with a significantly lower risk of spinal cord ischemia. Therefore, we intentionally used a shorter stent length while performing the FET technique for ATAAD. A stent ≥ 15 cm or coverage extending to T8 or further should be avoided [21].

SINE is a problem in the follow-up period after FET in ATAAD [22]. In our study, d-SINE occurred in two patients requiring TEVAR. With a TEVAR procedure, the risk of additional therapy is low. In our study, the incidence of d-SINE was lower than that in other reports, potentially because of the shorter observation periods and avoidance of oversized FET devices. As the observation periods become longer, the incidence of d-SINE may increase. Ogino et al. reported that the FET device should be inserted in a straight position in the descending thoracic aorta and that oversized FET devices should be avoided to prevent SINE [23].

This study had some limitations. Firstly, it had a retrospective design. Secondly, the present study had a short observation period and included few cases. However, this is the first longitudinal observational study comparing AAD and TAR with FET; therefore, further studies with more extended observation periods and larger sample sizes are warranted. Multicenter studies are required to validate our findings.

## Conclusions

TAR with FET showed perioperative results comparable to AAR’s for acute DeBakey type I aortic dissection. This technique also had significantly higher rates of freedom from aorta-related events in both the 1 and 3-year follow-up periods. It was thus considered a valuable method to avoid aorta-related events, even in the midterm.

## Data Availability

The datasets generated or analyzed during the current study are included in this published article.

## Abbreviations

AAR: Ascending aortic replacement
ATAAD: Acute type A aortic dissection
AVR: Aortic valve replacement
BCA: Brachiocephalic artery
CABG: Coronary artery bypass grafting
CPB: Cardiopulmonary bypass
CTD: Connective tissue disease
d-SINE: Distal stent graft-induced new entry
FET: Frozen elephant trunk
HD: Hemodialysis
IQR: Interquartile range
LCA: Left carotid artery
LSA: Left subclavian artery
TAR: Total arch replacement
TEVAR: Thoracic endovascular aneurysm repair
